# Redesigning a faculty development program for clinical teachers in Indonesia: a before-and-after study

**DOI:** 10.3352/jeehp.2024.21.14

**Published:** 2024-06-13

**Authors:** Rita Mustika, Nadia Greviana, Dewi Anggraeni Kusumoningrum, Anyta Pinasthika

**Affiliations:** 1 Department of Medical Education, Faculty of Medicine, Universitas Indonesia, Jakarta, Indonesia; 2 Medical Education Collaboration Cluster, Indonesia Medical Education and Research Institute, Universitas Indonesia, Jakarta, Indonesia; Hallym University, Korea

**Keywords:** COVID-19, Feedback, Indonesia, Medical education, Teacher training

## Abstract

**Purpose:**

Faculty development (FD) is important to support teaching, including for clinical teachers. Faculty of Medicine Universitas Indonesia (FMUI) has conducted a clinical teacher training program developed by the medical education department since 2008, both for FMUI teachers and for those at other centers in Indonesia. However, participation is often challenging due to clinical, administrative, and research obligations. The coronavirus disease 2019 pandemic amplified the urge to transform this program. This study aimed to redesign and evaluate an FD program for clinical teachers that focuses on their needs and current situation.

**Methods:**

A 5-step design thinking framework (empathizing, defining, ideating, prototyping, and testing) was used with a pre/post-test design. Design thinking made it possible to develop a participant-focused program, while the pre/post-test design enabled an assessment of the program’s effectiveness.

**Results:**

Seven medical educationalists and 4 senior and 4 junior clinical teachers participated in a group discussion in the empathize phase of design thinking. The research team formed a prototype of a 3-day blended learning course, with an asynchronous component using the Moodle learning management system and a synchronous component using the Zoom platform. Pre-post-testing was done in 2 rounds, with 107 and 330 participants, respectively. Evaluations of the first round provided feedback for improving the prototype for the second round.

**Conclusion:**

Design thinking enabled an innovative-creative process of redesigning FD that emphasized participants’ needs. The pre/post-testing showed that the program was effective. Combining asynchronous and synchronous learning expands access and increases flexibility. This approach could also apply to other FD programs.

## Graphical abstract

[Fig f4-jeehp-21-14]

## Introduction

### Background/rationale

Excellent clinical teachers are characterized by a balance between being good clinicians who inspire students and being good teachers who provide support, create interactivity, and exciting teaching moments. Therefore, faculty development (FD) programs for clinical teachers focusing on cognitive and non-cognitive teaching skills are important [1]. However, due to the busy clinical environment, the lack of time has been one of the main barriers preventing clinicians from attending FD programs [2].

Systematic approaches should be considered in planning, implementing, and evaluating FD programs to ensure accountable practice standards [3]. Furthermore, in the current disrupted situation, technology could widen access and create more engaging just-in-time programs, as traditional FD approaches, such as workshops, seminars, and structured programs, may fail to provide on-demand programs that facilitate self-regulated learning [4].

Although there have been reports on the use of technology in FD, reports on the transformation process are currently limited [4]. As faculty developers need to be creative and innovative in engaging faculty members and designing FD programs that involve technology, a flexible structure and guide such as design thinking—a 5-phase methodology for creative problem-solving—may also help scaffold their creativity and guide them in dealing with currently relevant problems facing FD programs for clinical teachers [5].

Faculty of Medicine Universitas Indonesia (FMUI) has an established FD program developed by the Medical Education Department of FMUI. Clinical teacher training (CT) is an FD program conducted since 2008, consisting of a 4-day intensive in-person training. The main objectives are to provide clinical teachers with sufficient knowledge and skills in clinical teaching—that is, the basic principles of clinical teaching, teaching-learning methods, and assessment. Workshops, in the form of role-playing and discussions, are led by skilled trainers who are a combination of clinical teachers and medical educationalists.

Following the safety issues raised during the coronavirus disease 2019 (COVID-19) pandemic, which required everyone to be distanced physically, the use of virtual and online clinical teacher development programs was preferred. Considering the availability and accessibility of technology and reflecting upon the need to incorporate technology for future programs, faculty developers at FMUI also realized that following the pandemic, it was necessary to transform the CT program to reach more participants in remote teaching hospitals and medical education institutions.

### Objectives

This study aimed to redesign an existing FD program for clinical teachers and test the new program’s effectiveness. The study’s specific aims were as follows: first, to develop a prototype focusing on participants’ needs amidst the current situation and reflecting the creativity of the FD developers; and second, to test the program’s effectiveness.

## Methods

### Ethics statement

This study received ethical clearance from the FMUI Research Ethics Committee (KET-641/UN2.F1/ETIK/PPM.00.02/2022).

### Study design

The study was conducted in 2 steps. The first step aimed to redesign the final prototype of the program, and the second step was to test the program’s effectiveness. In redesigning the program, the Stanford 5-phase approach to design thinking, consisting of empathizing, defining, ideating, prototyping, and testing (https://web.stanford.edu/class/me113/d_thinking.html), was used. In the second step, we employed the single pre/post-test approach, which was described according to the TREND (Transparent Reporting of Evaluations with Nonrandomized Design).

### Setting

As one of the oldest medical schools in Indonesia, the FMUI has established a high standard of teaching-learning and FD processes. Hence, the Department of Medical Education at FMUI has been appointed and received demands from other public and private medical schools to conduct CT programs for clinical teachers in medical education institutions and teaching hospitals across Indonesia. The pre-existing CT program was a 4-day intensive in-person training with interactive lectures and workshop sessions each day. Workshops in role-playing sessions were conducted on essential skill sets of clinical teachers, such as giving constructive feedback, conducting micro-skills teaching, bedside teaching and procedural skills, and workplace-based assessments. Group discussions assisted by facilitators were also conducted on most topics.

### Participants

Seven medical educationalists, 4 senior clinical teachers, and 4 junior clinical teachers participated in the empathizing steps of design thinking. There were 2 iterative design thinking processes; thus, pre-post testing was done twice, once in each round of testing. A total of 437 participants participated in our FD program, with 107 participants in the first round of testing and 330 participants in the second round from various institutions in Indonesia.

### Intervention

The prototype developed through the design thinking process was a 3-day blended learning training program based on an existing traditional training program established offline. The program aimed to provide clinical teachers with the practical knowledge and skills for teaching in clinical settings. The topics included (1) principles of teaching in a clinical setting, consisting of self-reflection, clinical reasoning, and giving constructive feedback; (2) teaching methods in a clinical setting—namely, bedside teaching, micro-skill teaching, and teaching procedural skills; (3) workplace-based assessments, consisting of mini-clinical evaluation exercise, mini-peer assessment tool, case-based discussion, log-books, and portfolio assessments. Each topic required skill acquisition facilitated using role play, demonstration, and discussion.

### Outcomes

Participants were required to fill in a program evaluation questionnaire at the end of each day’s sessions regarding their views, reactions, and satisfaction with the program. Participants were also required to submit the pre- and post-tests, respectively, before and after the completion of each day in the online platform to evaluate learning progress.

### Data sources/measurement

The program evaluation questionnaire at the end of each day’s sessions consisted of 2 items with a 4-point Likert scale and 1 open-ended question (Supplement 1). Faculty developers designed pre- and post-tests according to the topics given each day, and the same sets of questions were used for both pre- and post-tests each day. Data from both measurements (4-point Likert scale on satisfaction and pre/post-tests) are available in the raw data files (Dataset 1).

### Bias

All participants were included in the pre/post-testing. Hence, there was no participant bias in this study.

### Study size

Total sampling was used in this study.

### Assignment method

This study used design thinking with a single pre/post-test approach; this study had no specific assignment.

### Blinding (masking)

There was no blinding in this study since this is a single-group study. Pre/post-tests were done using online platforms, and data were directly obtained from the platform.

### Unit analysis

All participants’ data were analyzed individually.

### Statistical methods

Testing was done in 2 rounds (first and second rounds of testing) involving clinical teachers from 4 and 6 institutions, respectively. We divided participants based on the locations of their institutions in Indonesia (Sumatra, Jakarta, Java, and Kalimantan area) for the quantitative analysis. The transformed program was evaluated using Kirkpatrick’s framework [6]. Quantitative and qualitative secondary data from participants’ questionnaires and submitted pre- and post-tests were used to evaluate the program. Descriptive content analysis was conducted using qualitative data from the questionnaire. Pre- and post-test data of participants were analyzed using the Wilcoxon test (to compare the pre-test and post-test inside the same group), the Mann-Whitney test (to compare between each round of testing), and the Kruskal-Wallis test (to compare between areas) as the data were not normally distributed. Quantitative data were analyzed using IBM SPSS for Windows ver. 22.0 (IBM Corp.).

## Results

### Participants

**Fig. 1.**
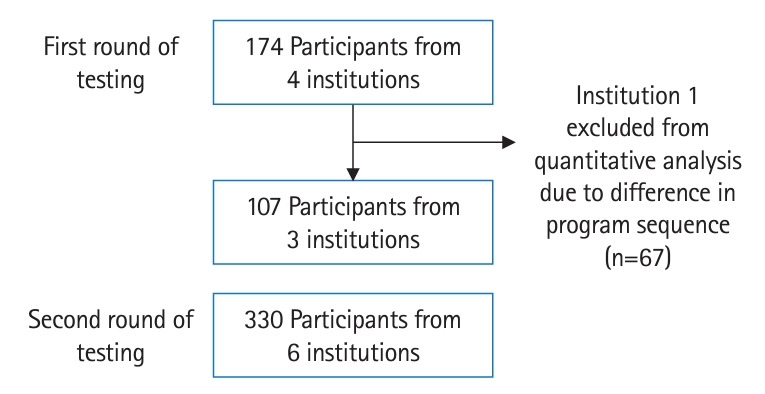
Flow of participants in the testing phase.

A total of 437 participants participated in our FD program, with 107 participants in the first round of testing and 330 participants in the second testing from various institutions in Indonesia (Table 1). Initially, 4 institutions were included in the testing, with 174 participants in the first round of testing. Later, we excluded Institution 1 from the quantitative analysis because we made major changes to the sequence of topics and synchronous session schedule of the prototype used for clinical teacher training at Institution 1 compared to other institutions (Fig. 1).

### Main results

**Fig. 2.**
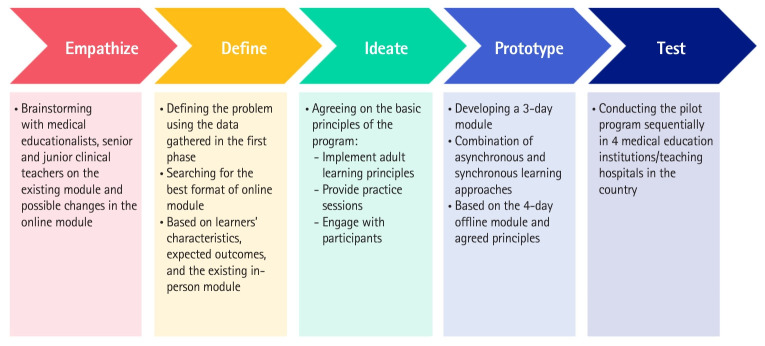
Phases of design thinking in program development.

We conducted 5-phase design thinking as described in Fig. 2. The topics covered in this new 3-day module were as follows: (1) day 1: the principles of clinical teaching; (2) day 2: the principles of teaching in clinical settings; and (3) day 3: the principles of assessment in clinical settings. The prototype was slightly adjusted for each training session in the testing phase, considering the feasibility, participants’ engagement, and the program evaluation results. The list of institutions participating in the testing can be viewed in Table 1 (Fig. 2).

### Results of the first round of testing

**Fig. 3.**
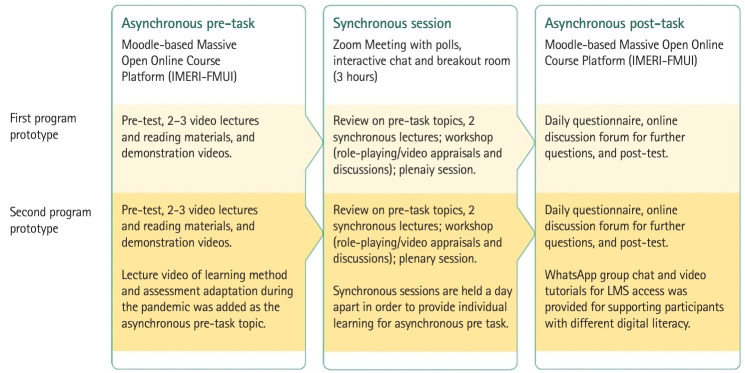
Daily learning sequence of the first and second program prototypes. IMERI, Indonesia Medical Education and Research Institute; FMUI, Faculty of Medicine Universitas Indonesia; LMS, learning management system.

Emerging themes on participants’ perceptions upon completing the first round of testing are presented in Table 2. We made several changes in the second program prototype based on the evaluations in the first round of testing, as illustrated in Fig. 3.

### Results of the second round of testing

Six institutions participated in the second round of testing with 330 participants (Table 1). We compared the participants’ perceptions regarding synchronous and asynchronous sessions between institutions in the first and second rounds of testing and between institutions’ areas (Sumatra, Jakarta, Java, and Kalimantan area) (Table 3). All the groups showed that most participants perceived both asynchronous and synchronous sessions positively. We found significantly more positive perceptions toward asynchronous and synchronous sessions among the second testing group compared to the first (P-value=0.003 and 0.008, respectively). Furthermore, the second testing group showed significantly more positive perceptions toward asynchronous sessions than toward synchronous sessions (P=0.002). We also observed that participants from the Sumatra area had more positive views towards asynchronous sessions than synchronous sessions (P=0.001).

The results of this study showed that participants from across areas in Indonesia had slightly higher prior knowledge (higher pre-test scores) in regard to the principles of clinical learning (day 1 topic) than for the principles of teaching and assessment in clinical settings (day 2 and 3 topics). We found a significant difference between pre-test and post-test scores among all groups, with higher post-test median scores for all sessions (P<0.001). We also observed significantly higher scores in the second testing group than in the first, except for the day 1 pre-and post-tests. There were no significant differences in pre-test scores across areas on the day 2 and day 3 topics. The participants from the Java and Kalimantan areas had significantly lower post-test scores for all days than those from other areas.

## Discussion

### Key results

The main aim of this study was to redesign an existing FD program for clinical teachers to develop an effective FD program suitable to the characteristics of clinical teachers and current situations. A final prototype of a 3-day blended learning FD program was developed through the 5-phase design thinking process. The program was deemed beneficial and suitable for participants because the design allowed participants to study the material through asynchronous platforms before synchronous sessions. Technical issues in the first testing were solved by providing technical support via a WhatsApp group. Providing specific time to study the material before the synchronous session solved the problem of finding time to study. The significant difference between the pre-test and post-test scores shows the knowledge gained through the program. The program also expanded access for participants from all over the country, and no significant difference was found in the increases in scores according to individuals’ places of origin.

### Interpretation

The CT program was redesigned following the COVID-19 pandemic, and the findings show that the combination of an asynchronous learning management system (LMS) for pre-task learning and synchronous (online or in-person) is still relevant in the post-pandemic era. Participants expressed positive attitudes toward asynchronous learning as it provides self-paced learning, in which participants can access the asynchronous materials at their convenience without interfering with patient care. Moreover, an online course provides various learning modalities (video, text, and H5P) that might cater to various learning styles. This perception may also be related to participants’ ability to demonstrate online self-regulated learning practice. This may align with a previous study in Indonesia showing that early-career medical doctors had a high online self-regulated learning ability in asynchronous learning [7]. These results demonstrated that an asynchronous approach is useful for expanding access to FD.

As completing this module required the efforts and self-regulated learning skills of participants, especially in navigating self-study sessions, the program was reinforced with access restrictions on the Moodle platform so that participants were obliged to finish the module sequentially. They had to complete the self-study section to proceed to the next sections and ultimately finish the module and acquire a completion certificate. This certificate was required for further recognition by the participants’ institutions, which might have served as an external source of motivation for the participants [8].

The program’s impact was evaluated based on level 1 (reaction) and level 2 (learning) of the Kirkpatrick evaluation model. On the reaction level, participants faced technical internet connection challenges despite the positive perceptions of the asynchronous component. This issue was solved by informing the institutions regarding the schedule of the synchronous sessions beforehand and advocating for the importance of dedicated time for participants to join the program to secure a more stable connection. Support provided by the technical team through social media (a WhatsApp group) was also helpful for participants with less experience operating the LMS and Zoom meeting platform. The importance of giving spare time to access asynchronous sessions was also highlighted by the evaluation. Hence, in the second prototype, additional access time was given, leading to a higher satisfaction level from participants.

An interesting finding was the significant difference in satisfaction with asynchronous activities between the first and second test participants. This may be related to the fact that the second test was administered after asynchronous learning became more familiar. It has been shown that self-efficacy, or the perception that one can easily navigate online learning, influences engagement and positive perception [9,10]. The significant increase in post-test scores across groups showed that the program could facilitate learning and knowledge attainment across periods and areas.

### Comparison with previous studies

The FD program involved curriculum design processes; thus, design thinking could be applied to address the user’s needs, as mentioned in a study by Anderson et al. [11]. The results of this study align with findings by Moon and D’Eon [12], showing how prioritizing the needs of faculty members in designing an FD program and incorporating technology-enhanced strategies could engage faculty and foster their self-directed learning. Furthermore, thoughtful and effective use of technology, such as combining synchronous and asynchronous sessions, helped participants to achieve learning objectives by integrating the flexibility of self-learning with the engagement and immediate feedback of online face-to-face sessions [13].

### Limitations

A single pre/post-test study was conducted without a control group; the program’s effectiveness would be better assessed with control and randomization.

### Generalizability

The FD program was developed using a design thinking approach that closely focused on participants’ specific needs and was tested on participants from several institutions from all over the country. Therefore, the rigorous process makes it generalizable to other institutions. However, because this is a single-institution study, caution is still needed.

### Suggestions

Given the flexibility of the design thinking approach, allowing more iteration processes to find the best prototype is crucial. Using the evaluation results to repeat the empathizing stage would help generate a better program, even after the second test. This study shows that the design thinking process would be suitable for designing other FD programs in the future. Moreover, pre/post-testing research would be beneficial in assessing program effectiveness.

### Conclusion

Design thinking is suitable for redesigning a participant and situation-focused FD program. It enhances creativity and balances the participant’s needs and the program’s goal. Technology usage is a participant-focused problem-solver for a program requiring flexibility for self-regulated learners as clinical teachers in this study.

## Figures and Tables

**Figure f4-jeehp-21-14:**
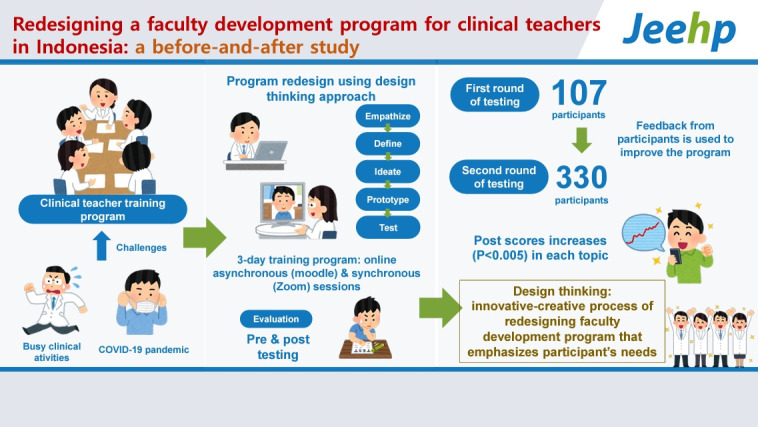


**Table 1.** Participating institutions in the test phase

Participating institutions	Area	No. of participants
First round of testing		
Institution 1 (teaching hospital)	Java & Kalimantan area	67
Institution 2	Jakarta area	40
Institution 3	Java & Kalimantan area	31
Institution 4 (teaching hospital)	Jakarta area	36
Total		174
Second round of testing		
Institution A	Jakarta area	37
Institution B (teaching hospital)	Jakarta area	52
Institution C	Jakarta area	54
Institution D	Java & Kalimantan area	39
Institution E	Sumatra area	42
Institution F	Sumatra area	106
Total		330

**Table 2.** Emerging themes of the first round of testing

Theme	Quotes
Challenges according to the first round of testing	
Technical issues (e.g., internet connection)	“The problems experienced in the first day of training were more technical problems related to unstable internet connections; fortunately, the problem was handled well by the committee.” (Institution 3, day 1 questionnaire)
Juggling clinical duties and the program	“Please take into account the time for self-study, considering that there are several mandatory activities such as clinical services on the same day.” (Institution 2, day 3 questionnaire)
Benefits according to the first round of testing	
Providing feedback and the right ‘mindset’	“The second day of training sessions was fun. The practice and role-play ran smoothly, and the plenary provided input on the best mindset and teaching patterns for students.” (Institution 3, day 2 questionnaire)
Skills in teaching and assessing students	“Very good explanation from the presenters. The group discussions were very helpful to understand how to assess students and fill out the (workplace-based) assessment forms.” (Institution 1, day 3 questionnaire)
Basic knowledge provided on asynchronous sessions	“The pre-test, video lectures, and workshop materials were well prepared by the committee and could be accessed and studied before the Zoom session, so we had good basic knowledge for participating in lectures and workshops on the first day. The implementation of the first day of training was good and on time; the workshop supervisor was very interactive and motivated us to participate in the workshop actively.” (Institution 3, day 1 questionnaire)

**Table 3.** Perceptions of synchronous and asynchronous sessions and the results of pretest and post-test based on testing and institutions area

	First round of testing (n=107)	Second round of testing (n=330)	P-value	Sumatra area (n=148)	Jakarta area (n=219)	Java & Kalimantan areas (n=70)	P-value
Asynchronous	3.5 (3-4)	3.89 (2.75–4)	0.003^*^	3.82 (2.75–4)	3.67 (2.87–4)	3.915 (2.89–4)	0.533
Synchronous	3.56 (2.78–4)	3.78 (2.83–4)	0.008^*^	3.67 (2.93–4)	3.67 (2.78–4)	3.725 (2.89–4)	0.602
P-value	0.873	0.002^*^		0.001^*^	0.487	0.828	
Day 1							
Pre-test	6 (2-8.670)	6 (1.33–10)	0.627	6 (1.33–10)	6 (2–9.33)	6 (2–8.67)	0.002^*^
Post-test	9.33 (2.67–10)	8 (1.33–10)	<0.001^*^	8 (1.33–10)	8.67 (2.67–10)	8 (2.67–10)	<0.001^*^
P-value	<0.001^*^	<0.001^*^		<0.001^*^	<0.001^*^	<0.001^*^	
Day 2							
Pre-test	4 (2–10)	5 (1–10)	0.023^*^	5 (1–10)	5 (2–10)	5 (1–10)	0.119
Post-test	8 (4–10)	9 (3–10)	0.019^*^	9 (3–10)	9 (3–10)	7 (3–10)	<0.001^*^
P-value	<0.001^*^	<0.001^*^		<0.001^*^	<0.001^*^	<0.001^*^	
Day 3							
Pre-test	4.55 (0.91–8.18)	5.450 (0–10)	0.001^*^	5 (0–10)	4.55 (0.91–10)	4.55 (0.91–8.18)	0.535
Post-test	7.27 (1.82–10)	8.18 (1.82–10)	<0.001^*^	8.18 (2.73–10)	7.27 (1.820–10)	6.36 (1.82–10)	0.004^*^
P-value	<0.001^*^	<0.001^*^		<0.001^*^	<0.001^*^	<0.001^*^	

Values are presented as median (range) unless otherwise stated.

* P<0.005 (Statistically significant).

## Footnotes

**Data availability**
Data files are available from Harvard Dataverse: https://doi.org/10.7910/DVN/NP6V35 Dataset 1. Raw data of participants’ responses to the questionnaires.

## Supplementary materials

Supplementary files are available from Harvard Dataverse: https://doi.org/10.7910/DVN/NP6V35

Supplement 1. Items from the evaluation questionnaire of the first pilot program.

Supplement 2. Audio recording of the abstract.
